# Long non-coding RNAs HERH-1 and HERH-4 facilitate cyclin A2 expression and accelerate cell cycle progression in advanced hepatocellular carcinoma

**DOI:** 10.1186/s12885-021-08714-7

**Published:** 2021-08-26

**Authors:** Tao Liu, Qiao Shi, Lei Yang, Shusen Wang, Hongli Song, Zhenglu Wang, Xinnv Xu, Hongsheng Liu, Hong Zheng, Zhongyang Shen

**Affiliations:** 1 National Health Commission’s Key Laboratory of Critical Care Medicine, Tianjin First Central Hospital, School of Medicine, Nankai University, No. 24 Fukang Road, Nankai District, Tianjin, 300192 China; 2 Key Laboratory of Transplant Medicine, Chinese Academy of Medical Sciences, Tianjin First Central Hospital, School of Medicine, Nankai University, Tianjin, 300192 China; 3 Graduate School, Tianjin University of Traditional Chinese Medicine, Tianjin, 301617 China; 4 Department of Clinical Laboratory, Tianjin First Central Hospital, School of Medicine, Nankai University, Tianjin, 300192 China; 5 Organ Transplant Center, Tianjin First Central Hospital, School of Medicine, Nankai University, Tianjin, 300192 China; 6 Biological Sample Resource Sharing Center (BSRSC), Tianjin First Central Hospital, School of Medicine, Nankai University, Tianjin, 300192 China

**Keywords:** Hepatocellular carcinoma, Cancer progression, Long non-coding RNA, Cell cycle

## Abstract

**Background:**

The advanced hepatocellular carcinoma (HCC), such as the recurrent tumor after liver transplantation (LT), is an obstacle of HCC treatment. The aim of this study was to discover the underlying mechanism of HCC progression caused by non-coding RNAs (ncRNAs).

**Methods:**

To this end, we investigated the selected patient cohort of matching primary and recurrent HCC after receiving LT. The recurrent tumors after LT were regarded as clinical models of the advanced HCC. Microarrays were used to profile lncRNA and mRNA expression in HCC recurrent and primary tissue samples. The mRNA profile characteristics were analyzed by bioinformatics. Two cell lines, HepG2 and QGY-7703, were used as HCC cell models. The protein-coding potential, length, and subcellular location of the interested lncRNAs were examined by bioinformatics, Northern blot, fluorescent in situ hybridization (FISH), and quantitative RT-PCR (qRT-PCR) assays. HCC cell proliferation was detected by CCK-8, doubling time and proliferation marker gene quantitation assays. DNA replication during the cell cycle was measured by EdU/PI staining and flow cytometry analyses. Promoter activity was measured using a luciferase reporter assay. Interactions between DNA, RNA, and protein were examined by immunoprecipitation and pull-down assays. The miRNA-target regulation was validated by a fluorescent reporter assay.

**Results:**

Both lncRNA and mRNA profiles exhibited characteristic alterations in the recurrent tumor cells compared with the primary HCC. The mRNA profile in the HCC recurrent tissues, which served as model of advanced HCC, showed an aberrant cell cycle regulation. Two lncRNAs, the highly expressed lncRNA in recurrent HCC (HERH)-1 and HERH-4, were upregulated in the advanced HCC cells. HERH-1/4 enhanced proliferation and promoted DNA replication and G1-S transition during the cell cycle in HCC cells. HERH-1 interacted with the transcription factor CREB1. CREB1 enhanced cyclin A2 (CCNA2) transcription, depending on HERH-1-CREB1 interaction. HERH-4 acted as an miR-29b/c sponge to facilitate CCNA2 protein translation through a competing endogenous RNA (ceRNA) pathway.

**Conclusions:**

The oncogenic lncRNA HERH-1/4 promoted CCNA2 expression at the transcriptional and post-transcriptional levels and accelerated cell cycle progression in HCC cells. The HERH-1-CREB1-CCNA2 and HERH-4-miR-29b/c-CCNA2 axes served as molecular stimuli for HCC advance.

**Supplementary Information:**

The online version contains supplementary material available at 10.1186/s12885-021-08714-7.

## Background

Hepatocellular carcinoma (HCC) is now the second leading cause of cancer death worldwide [1]. Aggression and postoperative recurrence are significant characteristics of HCC [2], and treatment of the advanced HCC remains as a challenge [3]. Liver transplantation (LT) is a common curative approach for HCC treatment. However, about 8–16% of HCC patients undergoing LT developed tumor recurrence [4–6]. During HCC recurrence, the potentially residual tumor cells in the micrometastasis or in circulation can migrate to the graft liver or extrahepatic organs, locate, and finally form a recurrent tumor, indicating an intense progression of these cells. The overall survival time after HCC recurrence is only 12.97 months [4]. Therefore, the analysis of recurrent HCCs after LT can help to explore new mechanisms of advanced hepatocarcinogenesis in order to gain insights into tumor progression.

The factors that drive HCC cells to become more aggressive are the alteration of molecular characteristics, such as cancer genome and gene regulation [7, 8]. The evolution of molecular expression and phenotype of HCC cells can be regarded as tumor heterogeneity [7]. Non-coding RNAs (ncRNAs), which refer to the RNA transcripts that lack significant protein-coding potential, are found to mediate various cellular processes in cancers [9]. Long non-coding RNAs (lncRNAs) are ncRNA transcripts that are longer than 200 nucleotides (nt). By interacting with proteins, DNA and other RNA molecules, lncRNAs widely carry out their biological roles at epigenetic, transcriptional, and post-transcriptional levels [10–13]. Some lncRNAs have been identified to be related to HCC progression, such as tumor recurrence after LT [14–16]. MicroRNAs (miRNAs, miR) are another class of small ncRNAs that bind with other RNA transcripts, either mRNAs or other ncRNAs, to inhibit protein translation or lead to degradation of target RNAs [17]. High-throughput screening discovered several miRNAs that serve as biomarkers of tumor recurrence risk after LT [18, 19]. The levels of some miRNAs in circulation or in serum exosomes are also indicative of HCC recurrence risk [20, 21]. However, the reported ncRNAs were mostly regarded as biomarkers in HCC advance and recurrence, and the underlying molecular pathway has not been adequately elucidated.

In this study, we found that two lncRNAs, HERH-1 and HERH-4, were highly expressed in the advanced HCC cells. These two lncRNAs promoted HCC cell cycle progression by facilitating CCNA2 expression, which may sequentially accelerate HCC progression.

## Methods

### Clinical tissue samples

Twelve pairs of human HCC tissue samples, including HCC primary and recurrent tissues from patients undergoing liver transplantation (Table S1), were obtained from the Biological Sample Resource Sharing Center (BSRSC) of the Tianjin First Central Hospital with the patients’ informed consent. After surgical resection or biopsy, the tissue samples were flash-frozen in liquid nitrogen and then stored at − 80 °C until use. Two pairs of the tissues were applied in microarray analysis, and the other ten pairs were used in the following quantitative reverse transcription PCR (qRT-PCR) for validation of the dysregulated lncRNAs. This study was performed in accordance with the Declaration of Helsinki and was approved by the Ethics Committee of the Tianjin First Central Hospital.

### HCC cell lines and transfection

An immortalized human benign hepatocyte cell line HL-7702, and four human HCC cell lines HepG2, QGY-7703, SMMC-7721 and Huh-7 were obtained from the Cell Bank of the Chinese Academy of Sciences (Shanghai, China) with confirmed identities of these cell lines. These cell lines were maintained in DMEM (Solarbio, Beijing, China; for HL-7702, HepG2 and Huh-7) or RPMI-1640 medium (Solarbio; for QGY-7703 and SMMC-7721) supplemented with 10% fetal bovine serum (FBS; Biowest, Nuaillé, France) at 37 °C in a humidified chamber supplemented with 5% CO_2_. Transfection of plasmids or oligonucleotides was performed using Lipofectamine 2000 (ThermoFisher, Waltham, MA, USA).

### RNA extraction

RNA was extracted from HCC tissues and cell lines using the *mir*Vana™ miRNA Isolation Kit (ThermoFisher), according to the manufacturer’s instructions. Long (> 200 nt) and short (< 200 nt) RNAs were isolated and purified. The separation of cytoplasmic and nucleic RNAs was achieved using a Cytoplasmic and Nuclear RNA Purification Kit (Norgen, Thorold, ON, Canada).

### Microarray analysis

Complementary DNA (cDNA) labeled with Cy3-dCTP (for primary HCC) or Cy5-dCTP (for recurrent HCC) was produced by Eberwine’s linear RNA amplification method and subsequent enzymatic reaction using a cRNA Amplification and Labeling Kit (CapitalBio, Beijing, China) [22]. The labeled cDNA was hybridized with CapitalBio Technology Human LncRNA Array V4 containing probes inspecting about 41,000 human lncRNAs and approximately 34,000 human mRNAs. Microarray data were analyzed using the GeneSpring software V13.0 (Agilent, Santa Clara, CA, USA). Genes with an absolute fold change value ≥2 and a Benjamini-Hochberg corrected *P*- value ≤0.05 were treated as differentially expressed genes. Hierarchical clustering analysis was performed using Cluster 3.0 software (Stanford University, CA, USA).

### qRT-PCR

For quantification of lncRNAs and protein-coding genes, 5 μg of long RNA was reverse transcribed into cDNA using oligo dT (for mRNAs) or Random 6 (for lncRNAs) primers using a PrimeScript II 1st Strand cDNA Synthesis Kit (TaKaRa, Otsu, Shiga, Japan). The cDNA was then used for amplification of the target RNA. β-actin was used as an endogenous control. Quantification of miRNAs and endogenous control U6 snRNA was performed using the stem-loop RT-PCR method [23]. All the qRT-PCR tests were carried out in two independent experiments with each PCR reaction performed in duplicate. The sequence of all primers and oligonucleotides used in this study are provided in Table S2.

All the real-time quantitative PCRs were performed using TB Green *Premix Ex Taq* II (TaKaRa) on a LightCycler 96 Real-Time PCR System (Roche, Basel, Switzerland). Gene expression was analyzed using LightCycler 96 software (V1.1, Roche).

### Northern blot assay

The lncRNA length was evaluated by Northern blot assay using a NorthernMax Kit (ThermoFisher), following the manufacturer’s instructions. The blot images were captured using a ChemiDoc XRS+ imaging system (Bio-Rad, Hercules, CA, USA).

### Fluorescent in situ hybridization (FISH) assay

The subcellular localization of lncRNA was evaluated by FISH assay using a Ribo Fluorescent In Situ Hybridization Kit (Ribobio, Guangzhou, China). Briefly, HCC cells were planted into 24-well plate at 8 × 10^4^ (HepG2) or 4 × 10^4^ (QGY-7703) per well. The transfected HCC cells were fixed in 4% paraformaldehyde and incubated in 0.5% Triton X-100 for higher membrane permeability. After prehybridization, the cells were incubated with Ribo lncRNA FISH Probe Mix (Ribobio) at 37 °C overnight. After stringency washing and DAPI staining, fluorescence was observed under an IX71 fluorescence microscope (Olympus, Shinjuku, Japan).

### Bioinformatics analyses

The protein-coding potential was assessed using the ORFfinder (https://www.ncbi.nlm.nih.gov/orffinder/) and CPAT (http://lilab.research.bcm.edu/cpat/index.php) databases. The concordance of HCC gene profiles from microarray data with defined gene sets was analyzed using the Gene Set Enrichment Analysis (GSEA) database (https://www.gsea-msigdb.org/gsea/index.jsp) [24]. Potential TFs or miRNAs binding with lncRNA were predicted using the RegRNA 2.0 database (http://regrna2.mbc.nctu.edu.tw/detection.html) [25]. The gene promoter region was predicted using PROMO (http://alggen.lsi.upc.es/cgi-bin/promo_v3/promo/promoinit.cgi?dirDB=TF_8.3) [26], and potential TFs binding with the gene promoter were predicted using PROMO and GeneCards (https://www.genecards.org/) [27]. The DNA-binding preferences of TFs were analyzed by JASPAR (http://jaspar.genereg.net/) [28]. Potential targets of miRNA were predicted using the TargetScanHuman database (http://www.targetscan.org/) [29].

### Artificial alteration of lncRNAs, miRNAs, and protein-coding genes in HCC cells

Overexpression of lncRNAs in HCC cells was achieved by the eukaryotic expression plasmid pcDNA3.1(+) (ThermoFisher). The exon fragments were amplified by PCR using human genomic DNA as a template. The fragments were then cloned into the pcDNA3.1(+) plasmid (Fig. S1). Suppression of endogenous lncRNAs was achieved by transfecting synthesized siRNA into HCC cells.

A double-strand RNA fragment was synthesized to serve as mimic of the miR-29b/c. A 2′-O-methyl modified single-strand RNA fragment that was inversely complementary to mature miR-29b/c (antisense oligonucleotide, ASO) was synthesized to serve as miR-29b/c inhibitor. These oligonucleotides were induced into HCC cells to artificially change miR-29b/c levels.

Overexpression of protein-coding genes was also achieved using the pcDNA3.1(+) vector. The full-length coding sequence was amplified by PCR using a human cDNA library as a template. The fragment was then cloned into the pcDNA3.1(+) plasmid.

### Cell counting kit-8 (CCK-8) cell viability assay

HCC cells were planted into 24-well plate at 8 × 10^4^ (HepG2) or 4 × 10^4^ (QGY-7703) per well and transfected on the next day. At 24 h after transfection, the cells were dissociated and planted into 96-well plate at 1 × 10^4^ (HepG2) or 5 × 10^3^ (QGY-7703) per well with three duplicates for each group. All the CCK-8 tests were carried out in three independent experiments. HCC cell viability was detected using the CCK-8 reagent (Dojindo, Tokyo, Japan). The absorbance at 450 nm (A_450_) was measured using an EnSpire Multimode Plate Reader (PerkinElmer, Waltham, MA, USA).

### Cell doubling time detection

The HCC cells were planted and treated similarly as the CCK-8 assay. At 24 h and 48 h after planting, the number of cells in each well of the 96-well plate was accurately counted. The doubling time was calculated using the formula: doubling time = Δt(lg2/(lgNt-lgN0)), in which Δt is the interval between the cell counting (24 h in this experiment), and N0 and Nt are the cell numbers at 24 h and 48 h after planting, respectively. All the cell doubling time tests were carried out in three independent experiments with each group in triplicate.

### 5-Ethynyl-2′-deoxyuridine (EdU) cell proliferation assays

As a kind of thymidine analog, EdU incorporates into genome DNA in the S phase during DNA synthesis. The quantity of the incorporated EdU reflects cell proliferation activity. EdU staining of HCC cells was performed using the Cell-Light EdU Apollo488 In Vitro Flow Cytometry Kit or Cell-Light EdU Apollo488 In Vitro Kit (Ribobio). For the flow cytometry analysis, HCC cells were planted into 6-well plate at 3 × 10^5^ (HepG2) or 1.5 × 10^5^ (QGY-7703) per well. The cells were transfected, stained and analyzed using an Accuri C6 Plus flow cytometer (BD, San Jose, CA, USA). For imaging, the HCC cells were planted and treated similarly as the CCK-8 assay. The cells in 96-well plate were stained and captured under an IX71 fluorescence microscope (Olympus). Fluorescence intensity was quantified using ImageJ software. The EdU cytometry and imaging tests were performed in two independent experiments with each group in duplicate.

### Propidium iodide (PI) staining cell cycle analysis

HCC cells were planted into 6-well plate at 3 × 10^5^ (HepG2) or 1.5 × 10^5^ (QGY-7703) per well. Approximately 10^6^ transfected HCC cells were fixed in 70% ethanol for at least 2 h. After washing with PBS, cells were stained in 1 mL PI staining solution containing 10 μg/mL of PI, 100 μg/mL of RNase A, and 0.1% Triton X-100 dissolved in PBS, for 30 min in the dark. Cell cycle distribution was analyzed by flow cytometry using an Accuri C6 Plus flow cytometer (BD). The cell cycle tests were performed in two independent experiments with each group in duplicate.

### Gene promoter efficiency analysis

The predicted CCNA2 promoter region was amplified by PCR and cloned into the pGL3/Enhancer vector (Promega, Madison, WI, USA). In addition, a CCNA2 promoter with deleted CREB1 binding sites was also amplified and cloned into the reporter vector. HCC cells were planted into 24-well plate at 8 × 10^4^ (HepG2) or 4 × 10^4^ (QGY-7703) per well. The reporter plasmids were transfected into HCC cells and the promoter efficiency was evaluated by measuring the luciferase activity using a Luciferase Assay System (Promega). Chemiluminescence was measured using an EnSpire Multimode Plate Reader (PerkinElmer). The gene promoter efficiency tests were performed in three independent experiments with each group in triplicate.

### Chromatin immunoprecipitation (ChIP) assay

The interaction between TF and gene promoter was confirmed by ChIP assay using ab500 ChIP Kit (Abcam, Cambridge, UK) according to the manufacturer’s instructions. Approximately 1 × 10^6^ HCC cells were collected for each ChIP assay. The TF conjugated DNA fragments were purified and the target DNA was identified using quantitative PCR.

### RNA pull-down assay

The interaction between lncRNA and TF was detected by RNA pull-down assay using a Pierce Magnetic RNA-Protein Pull-Down Kit (ThermoFisher) according to the manufacturer’s instructions. Approximately 6 × 10^6^ HCC cells were collected for each RNA pull-down assay. The potential RNA-binding proteins were eluted and purified for the further Western blot analysis.

### Western blot assay

Protein samples were resolved on an SDS denaturing polyacrylamide gel and transferred onto a nitrocellulose membrane (Boster, Wuhan, China). The membrane was incubated with the primary antibody overnight at 4 °C. The membrane was then washed and incubated with horseradish peroxidase (HRP)-conjugated secondary antibody. After chemiluminescence, the bands were captured using a ChemiDoc XRS+ imaging system (Bio-Rad). The band intensity was quantified using AlphaView SA software V3.4.0 (ProteinSimple, San Jose, CA, USA).

### RNA immunoprecipitation (RIP) assay

RNA targets of RNA-binding proteins were identified using a Magna RIP RNA-Binding Protein Immunoprecipitation Kit (Millipore, Billerica, MA, USA), following the manufacturer’s instructions. Approximately 2 × 10^7^ HCC cells were collected for each RIP assay. RNA in the RBP immunoprecipitation was purified and the target RNA was analyzed via RT-PCR and agarose gel electrophoresis.

### miRNA-target fluorescent reporter assay

HCC cells were planted into 24-well plate at 8 × 10^4^ (HepG2) or 4 × 10^4^ (QGY-7703) per well. After 24 h, the cells were transfected with a GFP reporter vector along with associated plasmids or oligonucleotides. At 48 h after transfection, the cells were lysed with RIPA lysis buffer (Solarbio) and GFP intensity was measured using an EnSpire Multimode Plate Reader (PerkinElmer). The fluorescent reporter assays were performed in three individual experiments with each group in triplicate.

### Statistical analysis

All numerical values were recorded as mean ± standard deviation (SD). The hypothesis test for significance between two groups utilized the Student’s *t* test. For three or more groups, one-way analysis of variance (ANOVA) was applied, followed by the Student-Newman-Keuls *q* test for comparing two of these groups. Statistical significance was set at *P* ≤ 0.05. Data processing and figure drawing were performed using a GraphPad Prism 7 software (GraphPad Software, La Jolla, CA, USA).

## Results

### HERH-1 and HERH-4 were expressed at a higher level in the advanced HCC

**Fig. 1:**
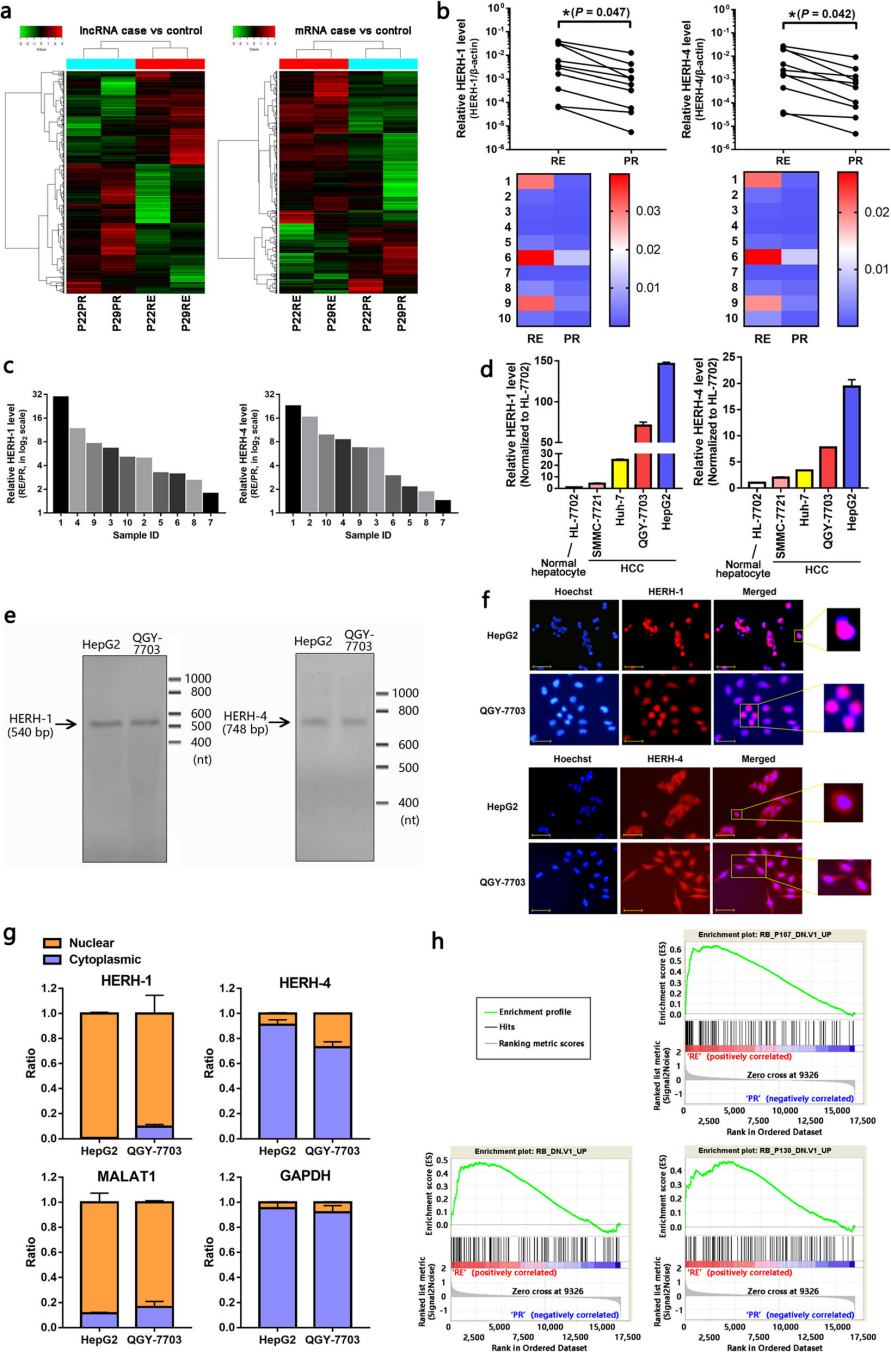
lncRNAs HERH-1/4 exhibited higher levels in radvanced HCC cells. **a** Cluster analysis of the lncRNA and mRNA profiles based on microarray data from two pairs of HCC recurrent (RE) and primary (PR) tissues in order to evaluate the significance of RNA profile on different tissue types. **b, c** The level of the two selected lncRNAs, HERH-1 and HERH-4, were examined in additional ten pairs of HCC recurrent (RE) and primary (PR) tissues by qRT-PCR to validate their level alteration. **d** In order to know the lncRNA level in HCC cell lines, the relative HERH-1/4 level in an immortalized benign hepatocyte cell line HL-7702 and four HCC cell lines were detected by qRT-PCR (*n* = 2). The lncRNA level in HL-7702 cells was set to 1. **e** The existence and general length of HERH-1/4 in HCC cells were detected by Northern blot. **f, g** The subcellular location of HERH-1/4 in HCC cells were detected by FISH (f) and qRT-PCR (g, *n* = 2) assays. Scale bar, 50 μm. **h** The gene signature of the HCC recurrent tissues was analyzed using the gene set enrichment analysis (GSEA) database. The three enrichment plots show the similarity of the recurrent HCC gene profile with the three RB-associated gene sets. *P < 0.05

The HCC recurrent tissues after LT were treated as typical clinical models of the advanced HCC. To assess the role of lncRNAs for advanced HCC, we investigated a patient sample set of 12 matching HCC primary and recurrent tissues by microarray and following qRT-PCR validation. The microarray analysis from two pairs of HCC tissues revealed that 716 lncRNAs and 2090 mRNAs were upregulated, and 997 lncRNAs and 958 mRNAs were downregulated in HCC recurrent tissues compared with the associated primary tumor tissues (Fig. S2; GEO accession: GSE102759). The cluster analysis indicated that the four HCC tissues could be grouped into two clusters as HCC recurrent and primary tissues (Fig. 1a). We selected the top five mostly dysregulated lncRNAs that have been noted in the Ensembl GRCh37 database (Table S3), and two of these lncRNAs showed good concordance with the microarray data by qRT-PCR in the other ten pairs of HCC tissues. We chose these two lncRNAs for further study, and named them as highly expressed lncRNAs in recurrent HCC (HERH)-1 and HERH-4 (Fig. 1b, c; Fig. S3; Table S4). Bioinformatics analysis indicated that HERH-1/4 lacked protein-coding potential (Table S5). HERH-1/4 was significantly induced in all the four human HCC cell lines compared to the immortalized, but benign hepatocyte cell line HL-7702, while HepG2 and QGY-7703 cells exhibited highest levels of HERH-1/4 (Fig. 1d). These two cell lines were applied in the following mechanistic studies.

Northern blot assays confirmed the existence of the two lncRNAs in HCC cells, and the length was in accordance with the prediction by the Ensembl GRCh37 database (Fig. 1e). More importantly, we found that HERH-1 was mainly located in the nucleus of HCC cells. HERH-4 appeared to be located in both the nucleus and the cytoplasm according to the FISH images. However, qRT-PCR assay confirmed that HERH-4 was mainly located in the cytoplasm of HCC cells (Fig. 1f, g). This indicated their possible functional patterns in the advanced HCC cells.

In order to determine the phenotypes that exhibited dominant alterations in the advanced HCC cells, we applied the GSEA database to analyze the mRNA profile (Table S6). Compared with the primary HCC, the mRNA expression pattern of the HCC recurrent tissues showed more similarity with that of the cells in which the RB gene was inhibited (Fig. 1h, Table S7). As a tumor suppressor, RB is a negative regulator of the cell cycle [30]. We presumed that the advanced HCC cells probably displayed an accelerated cell cycle progression.

### lncRNAs HERH-1/4 promoted HCC cell cycle progression

**Fig. 2:**
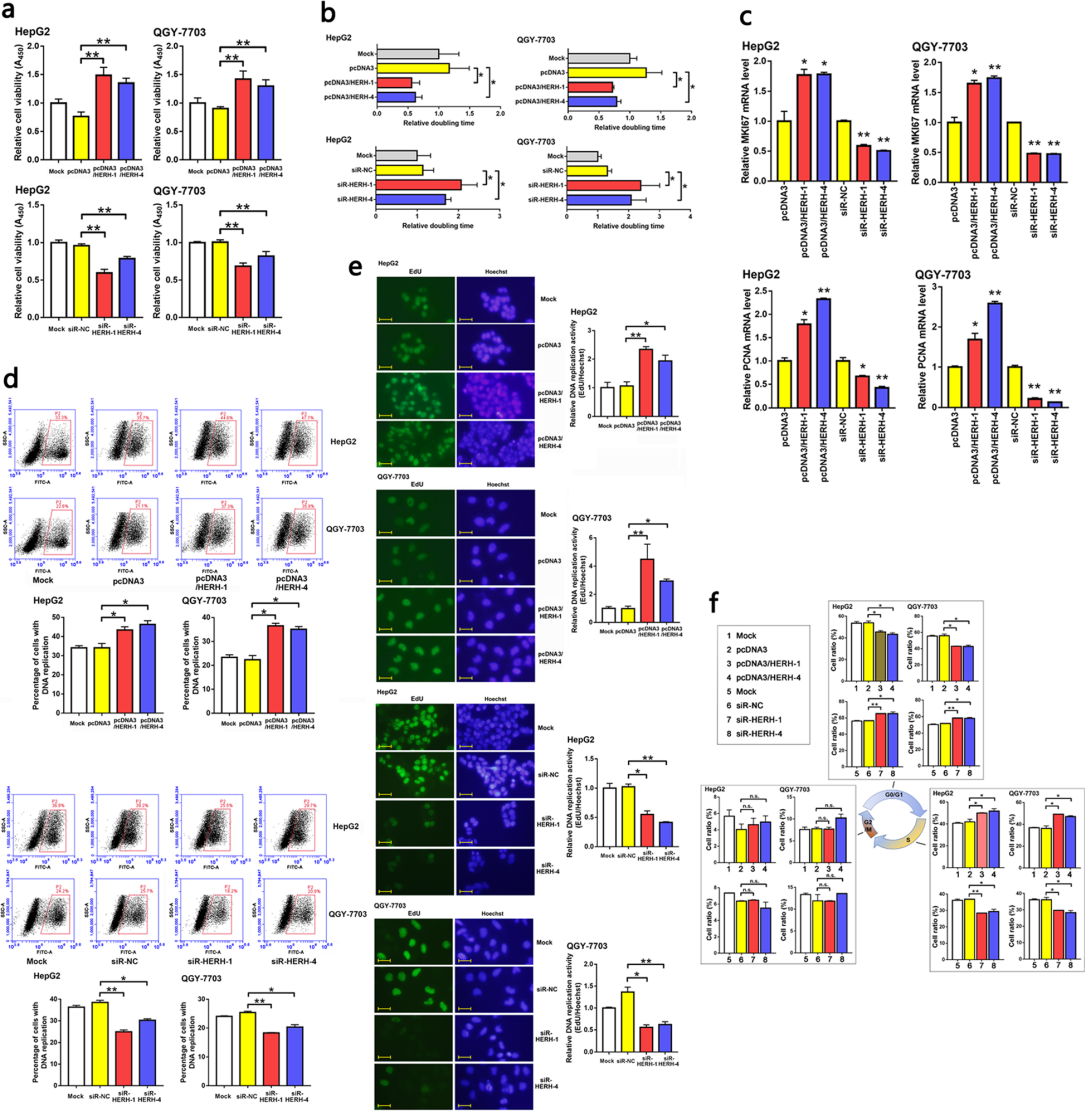
lncRNAs HERH-1/4 promoted HCC cell cycle progression. HERH-1/4 levels were artificially changed in HCC cells. **a** Cell viability affected by HERH-1/4 was detected by CCK-8 assay (*n* = 3). **b** The HCC cell proliferation rate affected by HERH-1/4 was detected by doubling time assay (*n* = 3). A shorter column indicates a faster proliferation. **c** The level of the two proliferation marker genes MKI67 and PCNA in HERH-1/4 altered HCC cells was measured by qRT-PCR (*n* = 2). **d, e** DNA replication in HCC cells was detected by EdU staining and flow cytometry (d, *n* = 2) or fluorescence microscopy (e, *n* = 2). Scale bar, 20 μm. **f** The HCC cell cycle distribution was detected by PI staining and flow cytometry analysis (*n* = 2). *P < 0.05; **P < 0.01

We used pcDNA3 eukaryotic expression vectors (Fig. S1) and small interfering RNAs (siRNAs) to achieve gain-of-function and loss-of-function of the two lncRNAs, respectively, in HCC cell lines (Fig. S4). Both HERH-1 and HERH-4 enhanced the viability and shortened the doubling time of HepG2 and QGY-7703 cells (Fig. 2a, b). MKI67 is a marker for general cell cycle activity and is expressed through G1–M phase. PCNA is expressed in cells undergoing DNA replication during S phase or DNA repair processes. The levels of these two proliferation marker genes also indicated positive regulation of these two lncRNAs on the proliferation of HCC cells (Fig. 2c). Genome DNA replication is a key process during cell proliferation. EdU cell proliferation assays suggested that HERH-1/4 levels were positively associated with the DNA replication rate (Fig. 2d, e). Furthermore, HERH-1/4 also affected the HCC cell cycle distribution. These two lncRNAs reduced HCC cells in the G1 phase, and increased those in the S phase, indicating a promoted G1-S transition. The number of cells in the G2/M phase was not affected by the lncRNAs (Fig. 2f). These data indicated that induced HERH-1/4 expression is associated with enhanced cell cycle and S phase activity.

### HERH-1 bound with CREB1 and facilitated CREB1-mediated CCNA2 transcription enhancement

**Fig. 3:**
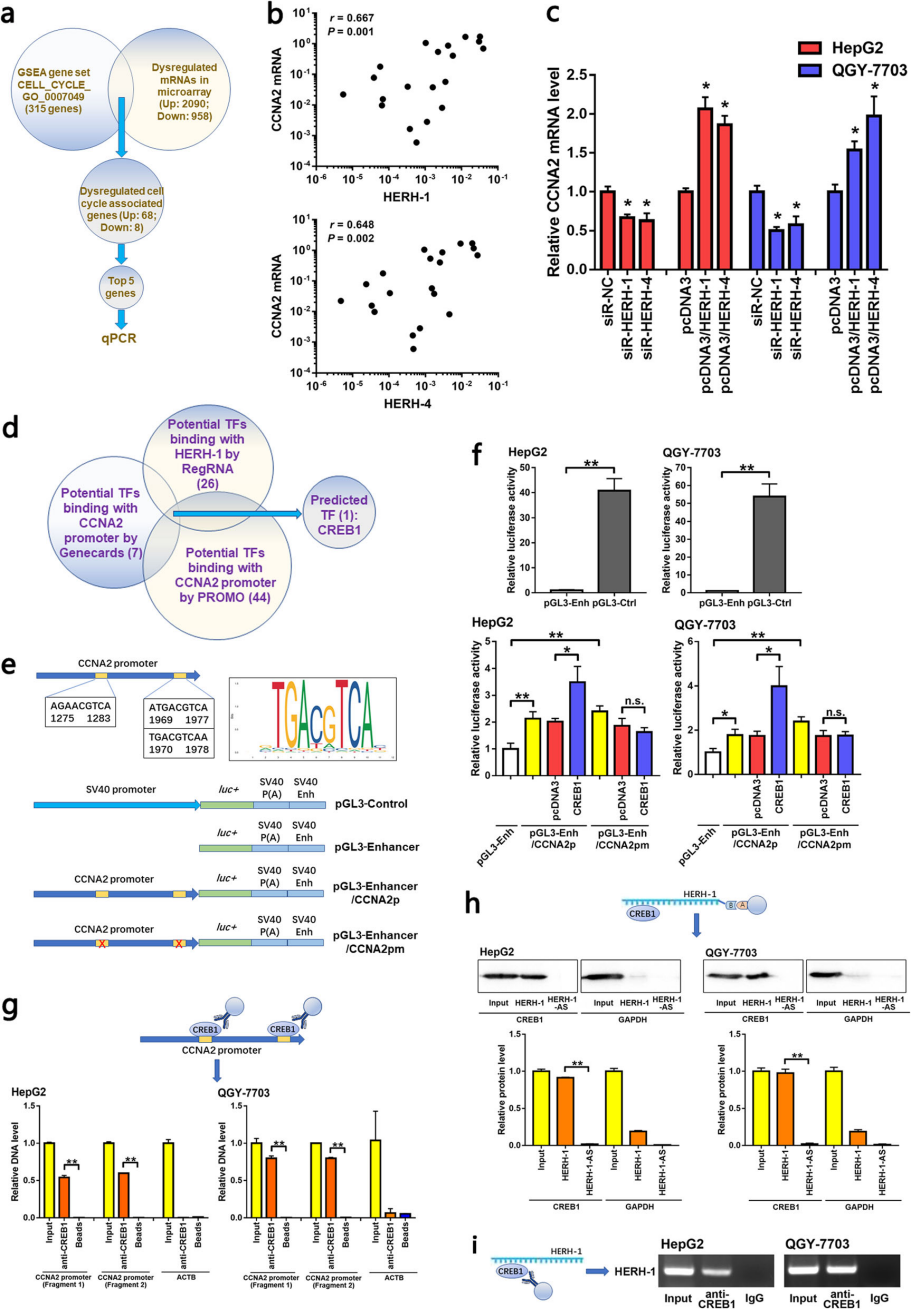
HERH-1 bound with CREB1 and facilitated CREB1-mediated CCNA2 transcription enhancement. **a** Through bioinformatics method, we found 315 cell cycle associated genes noted by the GSEA database. By combining this gene set with our microarray data, we screened out the top five significantly dysregulated cell cycle-associated genes in the advanced HCC, and part of these genes may be regulated by HERH-1/4. Among them, CCNA2 was the selected gene in the following study. **b** Correlation between HERH-1/4 and CCNA2 mRNA levels was analyzed in the ten pairs of HCC recurrent and primary tissues by qRT-PCR. **c** Regulation of CCNA2 by HERH-1/4 in HCC cells was validated by qRT-PCR (*n* = 2). **d** Potential TFs that can bind with HERH-1 and the CCNA2 promoter were predicted by bioinformatics. **e** Schematic presentation of the three predicted CREB1 binding sites within CCNA2 promoter (top) and the components of the plasmids used for promoter activity assessment (bottom). **f** Activity of the predicted CCNA2 promoter and the effect of CREB1 on transcription intensity was detected by luciferase reporter assay (*n* = 3). **g** The interaction between the CREB1 protein and CCNA2 promoter was detected by ChIP assay (*n* = 2). **h, i** The interaction between HERH-1 and CREB1 protein was detected by RNA pull-down (h) and RIP (i) assays. Full-length blots and gels are presented in Fig. S8a and Fig. S9a, b. *P < 0.05; **P < 0.01; n.s., not significant

Next, we tended to find out the functional protein-coding genes that were regulated by the lncRNAs HERH-1/4. It was presumed that the significant genes were probably involved in cell cycle process, and might be dysregulated in the advanced HCC cells. First, we searched the GSEA database and found that the gene set CELL_CYCLE_GO_0007049 records 315 genes annotated to the cell cycle (Table S8). Then, by combining our microarray data with this gene set, we found that 76 of the 315 cell cycle-associated genes were dysregulated in the microarray, in which 68 genes were upregulated and 8 genes were downregulated (Fig. 3a, Table S9). This method effectively reduced the number of the candidate genes. Among them, we chose the top five genes that showed the most obvious range of variation in the microarray, and detected their levels in HCC clinical tissues and cell lines. These genes had high probability to be regulated by HERH-1/4. As a result, cyclin A2 (CCNA2) levels exhibited a positive correlation with HERH-1/4 levels in the HCC recurrent versus primary tissues, and was found to be co-regulated in HCC cell lines (Fig. 3b, c). None of the other genes showed statistically significant correlation with the two lncRNAs (Fig. S5). These data indicated that CCNA2 was a potential target of HERH-1/4. Therefore, we investigated a potential regulation of CCNA2 by HERH-1/4.

Given that HERH-1 is mainly located in the nucleus, we considered that HERH-1 may regulate CCNA2 at the transcriptional level by interacting with transcription factors (TFs). According to RegRNA, 26 TFs may bind with HERH-1. PROMO and GeneCards predicted 44 and 7 TFs that potentially interact with the CCNA2 promoter, respectively (Table S10). CREB1 was the only TF that presented in all the three lists (Fig. 3d). In this study, we focused on the interaction of CREB1 with HERH-1 and the CCNA2 promoter.

There are three potential binding motifs within the CCNA2 promoter. In order to investigate the activity of the CCNA2 promoter, we introduced a luciferase reporter pGL3/Enhancer, in which the objective promoter sequence can be cloned at upstream of the luciferase coding region. A naive CCNA2 promoter (Table S11) or a mutated CCNA2 promoter lacking the three TF-binding sites for CREB1 were cloned into the pGL3/Enhancer plasmid (Fig. 3e). As a positive control, luciferase was expressed under control of the SV40 promoter (pGL3-Control; Fig. 3e, f). The naive CCNA2 promoter boosted luciferase expression in HCC cells. In a next step, we co-expressed CREB1 in these cells (Fig. S6) and found a further increase of the luciferase activity. Importantly, when the three potential binding sites of CREB1 were deleted, luciferase expression was no longer strengthened by CREB1 (Fig. 3f).

Next, we verified the interaction between CREB1, CCNA2 promoter and HERH-1 in HCC cells. ChIP assay suggested that the CREB1 protein was directly bound to the CCNA2 promoter region (Fig. 3g). The interaction between HERH-1 and CREB1 was verified by RNA pull-down (Fig. 3h) and RIP (Fig. 3i) assays. These results suggest that lncRNA HERH-1 binds with CREB1 and may facilitate the interaction of CREB1 with the CCNA2 promoter region.

### CREB1 promoted CCNA2 expression and accelerated HCC cell cycle progression depending on HERH-1

**Fig. 4:**
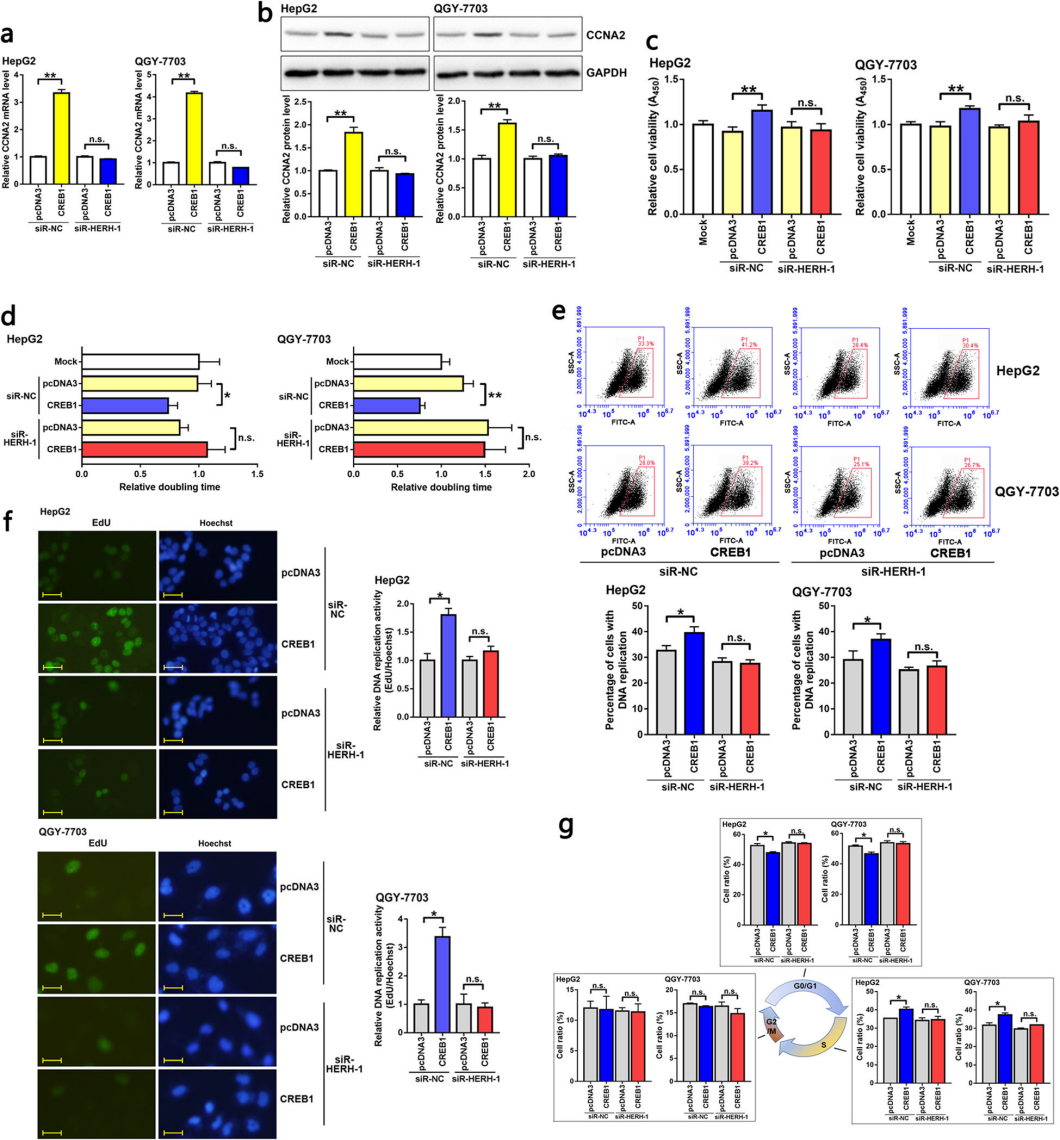
CREB1 promoted CCNA2 expression and enhanced HCC cell cycle progression depending on HERH-1. CREB1 was overexpressed in HCC cells with or without HERH-1 inhibition, and phenotypes of the HCC cells were detected to confirm the dependence of CREB1-assisted CCNA2 expression and function on HERH-1. **a, b** The endogenous CCNA2 level was measured by qRT-PCR (a, *n* = 2) and Western blot (b) assays. Full-length blots are presented in Fig. S8b. **c, d** The HCC cell viability and proliferation rate were detected by CCK-8 (c, *n* = 3) and doubling time (d, *n* = 3) assays. **e, f** DNA replication in HCC cells was detected by EdU staining and flow cytometry (e, *n* = 2) or fluorescence microscopy (f, *n* = 2). Scale bar, 20 μm. **g** The HCC cell cycle distribution was detected by PI staining and flow cytometry analysis (*n* = 2). *P < 0.05; **P < 0.01; n.s., not significant

In HCC cells, ectopic expression of CREB1 led to increased CCNA2 expression. This depended on the existence of HERH-1 because CREB1 failed to influence CCNA2 if endogenous HERH-1 was knocked down (Fig. 4a, b). As predicted, CREB1 enhanced cell viability (Fig. 4c), shortened the cell doubling time (Fig. 4d), accelerated genome DNA replication (Fig. 4e, f), and promoted the transition of cells from the G1 phase to the S phase (Fig. 4g) in HCC cell lines. In addition, when endogenous HERH-1 was blocked, the oncogenic effects of the CREB1 protein on HCC cells were no longer detectable (Fig. 4c–g). These results further demonstrated that HERH-1 facilitated CCNA2 expression by assisting CREB1 in CCNA2 transcription activity.

### HERH-4 acted as a miR-29b/c sponge to stabilize CCNA2 mRNA and assisted its translation

**Fig. 5:**
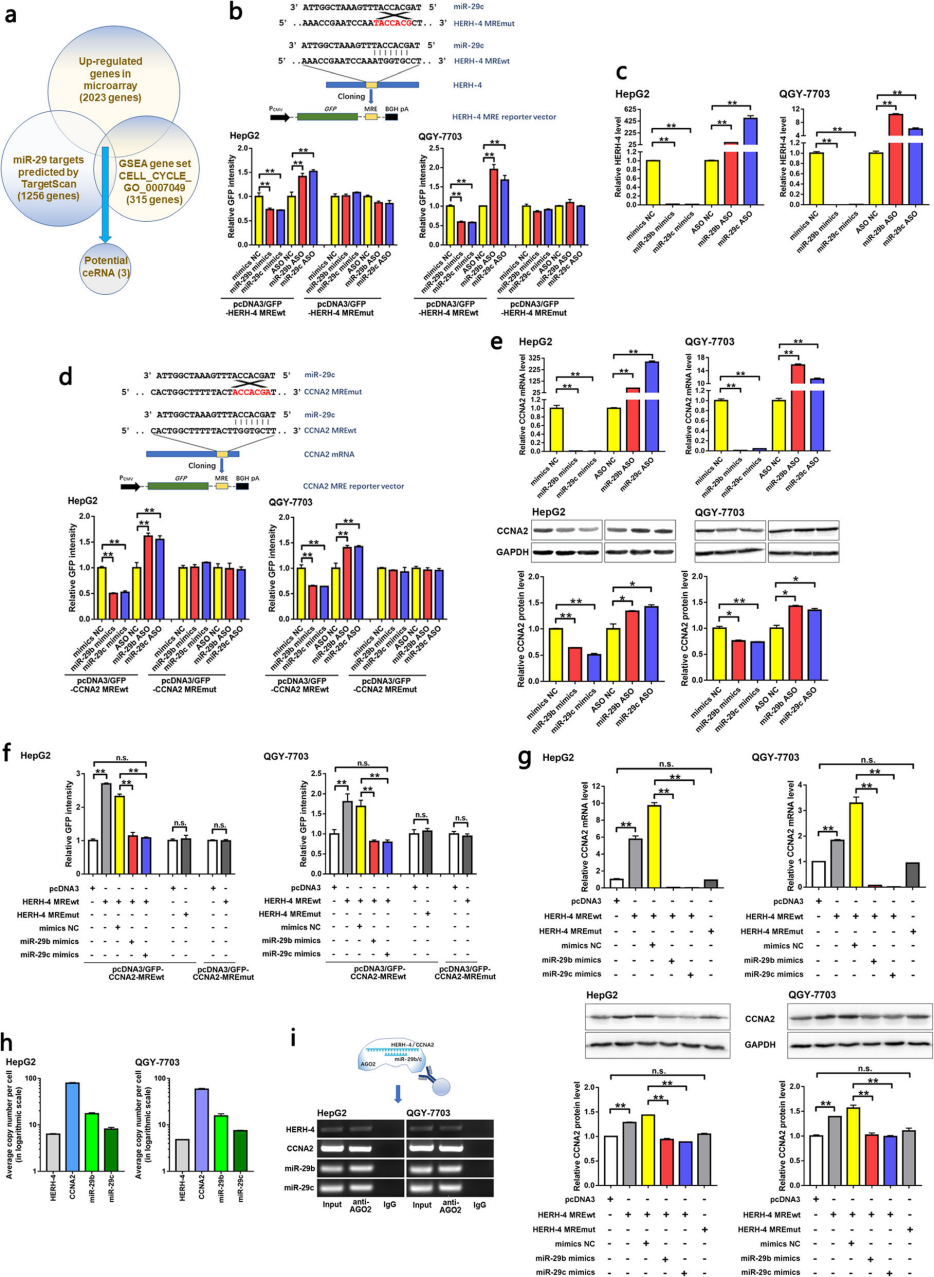
HERH-4 acted as a miR-29b/c sponge to stabilize CCNA2 mRNA and assisted its translation. **a** Potential ceRNA that bore the same MRE with HERH-4 was predicted by bioinformatics. We screened out the genes that were annotated as cell cycle associated genes by the GSEA database, showed upregulation in our microarray data, and were predicted as potential miR-29 targets by the TargetScan database. **b** The miR-29c response element within HERH-4 was cloned into a GFP reporter vector. The regulation of miR-29b/c on HERH-4 MRE function was detected by GFP reporter assay in HCC cells (*n* = 3). **c** To confirm the negative regulation of miR-29b/c on their target HERH-4, miR-29b/c levels were altered in HCC cells and the endogenous HERH-4 level was detected by qRT-PCR (*n* = 2). **d** To confirm the regulation of miR-29b/c on their target CCNA2, the miR-29c response element within CCNA2 mRNA was cloned into GFP reporter vector. The effects of miR-29b/c on the CCNA2 MRE function was detected by GFP reporter assay in HCC cells (*n* = 3). **e** The negative regulation of miR-29b/c on the endogenous CCNA2 level was detected by qRT-PCR (*n* = 2) and Western blot assays in HCC cells. Full-length blots are presented in Fig. S8c, d. **f, g** The positive regulation of HERH-4 MRE on CCNA2 expression and the role of miR-29b/c within the ceRNA regulation were detected by GFP reporter (f, *n* = 3), qRT-PCR (g, *n* = 2), and Western blot (g) assays in HCC cells. Full-length blots are presented in Fig. S8e-g. **h** The absolute level of HERH-4, CCNA2 mRNA, and miR-29b/c in HCC cells were detected by qRT-PCR (*n* = 2) in order to evaluate the molecular environment of the ceRNA regulation among these factors. **i** The recruitment of miR-29b/c, HERH-4 and CCNA2 mRNA to AGO2 protein was confirmed by RIP assay in order to support the fact that miR-29b/c enrolled their targets into RISC to exercise miRNAs’ function. Full-length gels are presented in Fig. S9c, d. ASO, antisense oligonucleotide; MRE, miRNA response element; NC, negative control; wt, wild-type; mut, mutant; *P < 0.05; **P < 0.01; n.s., not significant

Given that lncRNA HERH-4 is located in the cytoplasm, we explored its mechanism of HCC proliferation at the post-transcriptional level. According to the RegRNA 2.0 database, we found five miRNAs that possibly bound to HERH-4, and miR-29c-3p (miR-29c) had the highest score (Table S12). To search for the potential ceRNA of HERH-4, we used the TargetScan database to predict miR-29-3p targets, and used the GSEA database to identify the cell cycle-associated genes. In addition, we focused on the upregulated protein-coding genes in the microarray because a high HERH-4 level reasonably led to an increase in its ceRNA [31]. As a result, three genes, including CCNA2, were present in all these three gene sets (Fig. 5a; Table S13). The miR-29b is an analog of miR-29c, and they share the same seed region. The levels of miR-29b/c were artificially altered by introducing mimics or ASOs into HCC cells (Fig. S7). miR-29b/c suppressed HERH-4 level by interacting with the predicted miRNA response element (MRE; Fig. 5b). Endogenous HERH-4 was negatively regulated by miR-29b/c (Fig. 5c). Similarly, CCNA2 expression was directly suppressed by miR-29b/c (Fig. 5d, e). These data confirmed that both HERH-4 and CCNA2 mRNA were direct targets of miR-29b/c.

In order to validate that HERH-4 positively regulated CCNA2 expression via absorbing miR-29b/c, we overexpressed the miR-29b/c response elements within HERH-4 in HCC cells. We found the GFP reporter intensity to be increased with CCNA2 wild-type MRE, while further induction of miR-29b/c reduced the GFP intensity to basal level. The positive regulation of HERH-4 MRE on CCNA2 was undetectable when the sequence of either HERH-4 MRE or CCNA2 MRE was mutated (Fig. 5f). Endogenous CCNA2 expression was also regulated by HERH-4 MRE and miR-29b/c following the same model (Fig. 5g).

An optimal ceRNA cross-talk occurs at a near-equimolar equilibrium of all ceRNAs and miRNAs within a network [32]. Absolute quantification indicated that the four molecules involved in the ceRNA network had comparable levels in HCC cells (Fig. 5h). The RIP experiment demonstrated that all the four molecules were recruited to AGO2 protein (Fig. 5i), a key factor in the RNA-induced silencing complex in which miRNAs exert their function. These data further supported the ceRNA network between HERH-4 and CCNA2.

### HERH-4 promoted HCC cell cycle progression by absorbing miR-29b/c

**Fig. 6:**
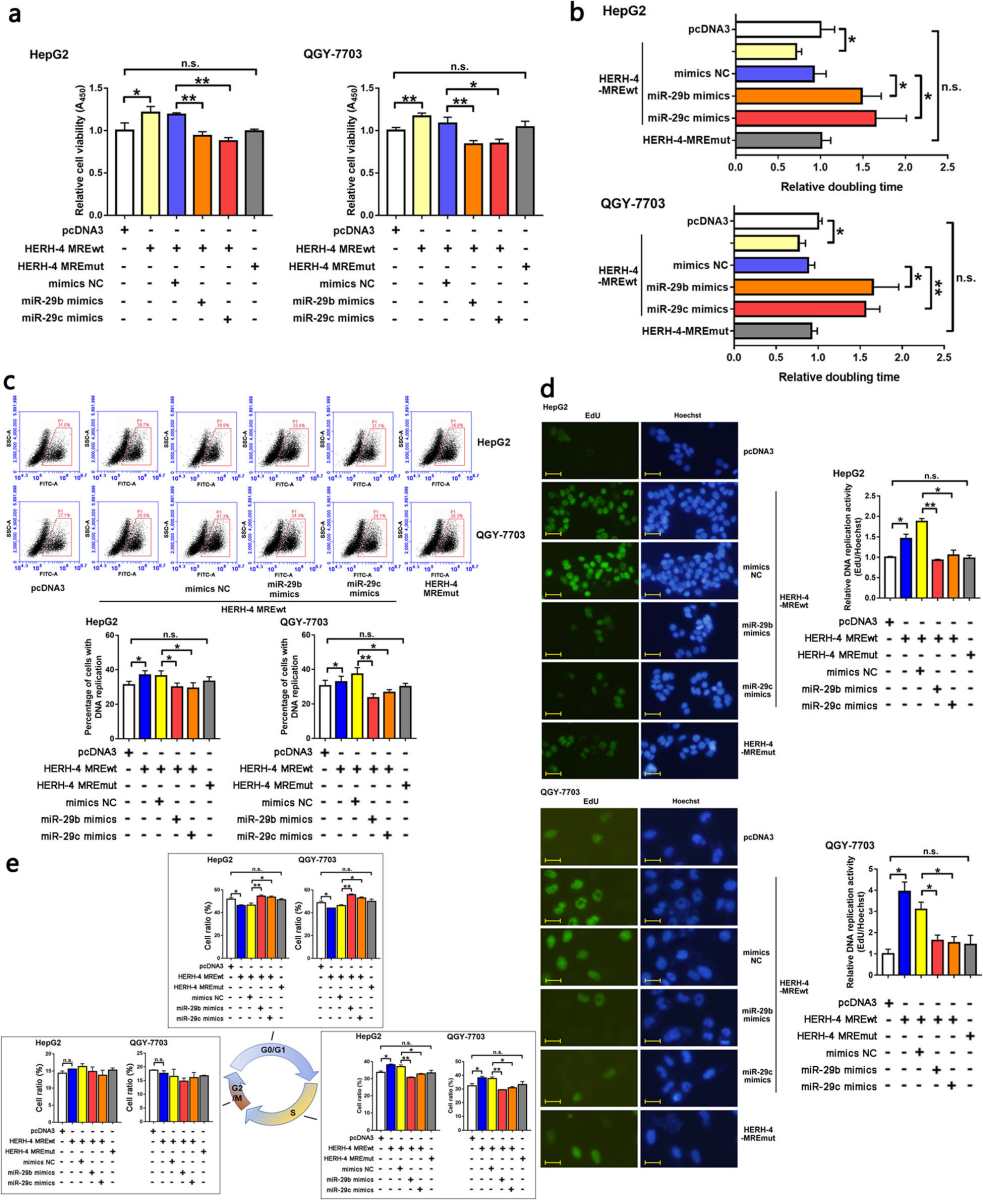
HERH-4 promoted HCC cell cycle progression by absorbing miR-29b/c. The HERH-4 MRE sequence was overexpressed, with or without transfection of miR-29b/c mimics in HCC cells in order to validate the ceRNA regulation of HERH-4 on CCNA2 and the following effects of cell cycle associated phenotypes in HCC cells. **a, b** The HCC cell viability and proliferation rate were detected by CCK-8 (a, *n* = 3) and doubling time (b, *n* = 3) assays. **c, d** DNA replication in HCC cells was detected by EdU staining and flow cytometry (c, *n* = 2) or fluorescence microscopy (d, *n* = 2). Scale bar, 20 μm. **e** The HCC cell cycle distribution was detected by PI staining and flow cytometry analysis (*n* = 2). MRE, miRNA response element; NC, negative control; wt, wild-type; mut, mutant; *P < 0.05; **P < 0.01; n.s., not significant

In order to analyze the effects of the HERH-4-miR-29b/c-CCNA2 ceRNA network on HCC progression, we overexpressed the 22 nt length of the miR-29b/c binding sequence within HERH-4, and found a higher viability (Fig. 6a), a shorter doubling time (Fig. 6b), and a rapider genome DNA replication (Fig. 6c, d) in HCC cell lines. The mutated MRE sequence failed to promote HCC cell proliferation (Fig. 6a–d). Importantly, if we introduced miR-29b/c mimics following overexpression of the wild-type HERH-4 MRE, the proliferation activity of the HCC cells restored (Fig. 6a–d). The wild-type rather than the mutated HERH-4 MRE reduced HCC cells in the G1 phase and increased those in the S phase, and miR-29b/c restored these effects (Fig. 6e). These data support the hypothesis that HERH-4 acts as a ceRNA to facilitate CCNA2 expression and promote HCC proliferation and cell cycle progression.

## Discussion

The high aggression rate has been a significant obstacle for HCC treatment [4–6]. The recurrent HCC after LT is characterized as significantly enhanced progression, making it a typical model of the advanced HCC cells. Recurrence of HCC is indicative for HCC advancing and metastasis, which indicates worse prognosis of the patients. Therefore, the study indented to illustrate the underlying molecular mechanism in HCC recurring.

Interestingly, more than 60% of the human genome is transcribed, and the protein-coding genes account for only less than 2% of the genome [33, 34]. The non-protein-coding transcripts are extensively involved in many cellular pathways and processes, including oncogenic signaling [34]. In this study, we obtained the following messages through microarray data. First, both mRNA and lncRNA profiles in the recurrent tumor cells exhibited characteristic changes compared with those in the HCC primary tissues. Second, we identified that a significant biological feature of the recurrent tumor cells was acceleration of the cell cycle progression. The retinoblastoma (RB) family plays a pivotal role in the negative control of the cell cycle and in tumor progression [30]. The advanced HCC cells exhibited a similar mRNA profile as the cells in which the RB gene family is downregulated, indicating a promoted cell cycle in these cells. Third, two of the obviously changed lncRNAs, named HERH-1 and HERH-4, were selected for mechanistic studies.

lncRNAs can interact with RNA-binding proteins (RBPs) and regulate their function [35]. These lncRNAs are required for the correct localization of the TFs to genome DNA [36]. In HCC cells, transcription of the cell cycle regulator CCNA2 was accelerated by CREB1, and this regulation process was HERH-1-dependent. This hypothesis was verified by the following experiments. First, CCNA2 transcription was accelerated by CREB1 protein, and this regulation depended on the three CREB1 binding motifs within the CCNA2 promoter. Second, HERH-1 directly interacted with CREB1 in a sequence-specific manner. Third, ectopic expression of CREB1 improved CCNA2 levels, promoted proliferation, and accelerated the cell cycle of HCC cells. Importantly, HERH-1 was essential in the CREB1-CCNA2 axis-mediated cell cycle acceleration. These data demonstrated that HERH-1 positively regulates CCNA2 expression and the HCC cell cycle at the transcriptional level.

lncRNA also acts as a negative regulator of miRNA [12, 13]. RNA transcripts can sequester a limited pool of special miRNAs and prevent other RNA molecules from being inhibited by these miRNAs, known as competing endogenous RNA (ceRNA) [31, 37, 38]. In HCC cells, the lncRNA HERH-4 acts as a natural miRNA decoy to promote CCNA2 expression at the post-transcriptional level. First, both HERH-4 and CCNA2 mRNA possess functional miR-29b/c binding sites. The miRNAs and their targets were all recruited to AGO2 protein, a key factor of RNA-induced silencing complex (RISC), in which the miRNA-targeted RNA molecules are degraded [39, 40]. Second, the MRE fragment within the HERH-4 sequence promoted CCNA2 gene expression, which was further restored by miR-29b/c mimics. Third, miR-29b/c and their two targets had comparable levels in HCC cells, which ensured an appropriate molecular environment for ceRNA cross-talk [32].

Cyclin is a classical protein family that controls cell cycle progression by activating cyclin-dependent kinase (CDK) enzymes or other cell cycle-associated factors. As a widely expressed cyclin A subtype, cyclin A2 (CCNA2) binds and activates CDK2 to control the G1/S transition [41]. Our research demonstrated two novel CCNA2 upstream regulation pathways involved in HCC progression. Another key regulator of G1/S transition cyclin E2 (CCNE2) was also upregulated in the advanced HCC (Table S9). The possible regulation of CCNE2 by HERH-1/4 are of importance to be explored in the future.

## Conclusions

**Fig. 7:**
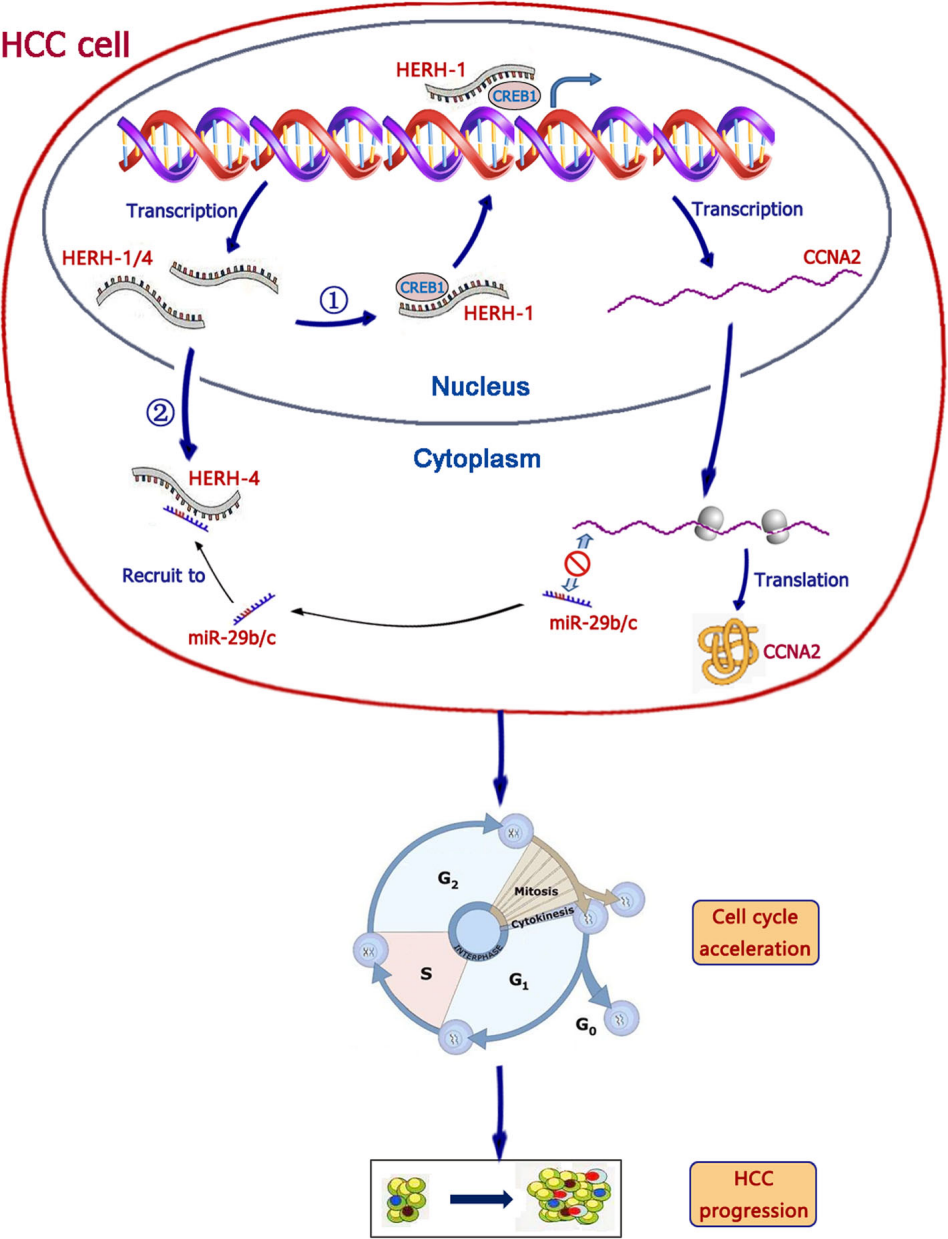
Schematic summary of lncRNAs HERH-1/4 mediated cell cycle acceleration in the advanced HCC cells. HERH-1 bound with the transcription factor CREB1 and facilitated its promotion on CCNA2 transcription. HERH-4 acted as a ceRNA to absorb miR-29b/c and shield CCNA2 mRNA from degradation. These two lncRNAs enhanced CCNA2 expression and accelerated HCC cell cycle progression. These pathways may be part of the mechanism of HCC progression

In summary, this study revealed two lncRNA-mediated cell cycle regulation pathways in the advanced HCC cells. During HCC evolution, the tumor cells exhibit accelerated cell cycle progression. The lncRNAs HERH-1/4 promoted HCC cell proliferation and the G1-S transition of the cell cycle. HERH-1 binds with CREB1 and assisted CCNA2 transcription. HERH-4 acted as an miR-29b/c decoy to alleviate the suppression of CCNA2 protein translation at the post-transcriptional level. The HERH-1/4-CCNA2 axis plays a crucial role during HCC progression (Fig. 7).

## Supplementary Information

**Additional file 1.**

**Additional file 2.**

## Data Availability

The RNA microarray data generated and analyzed during the current study are available in the Gene Expression Omnibus (GEO) repository (https://www.ncbi.nlm.nih.gov/geo/; accession: GSE102759). All data that support the findings of this study are available from the corresponding authors upon reasonable request.

## Abbreviations

AFP: Alpha-fetoprotein
ASO: Antisense oligonucleotide
CCK-8: Cell counting kit-8
CCNA2: Cyclin A2
ceRNA: Competing endogenous RNA
ChIP: Chromatin immunoprecipitation
CREB1: cAMP responsive element binding protein 1
CTC: Circulating tumor cell
EdU: 5-ethynyl-2′-deoxyuridine
FISH: Fluorescent in situ hybridization
GEO: Gene Expression Omnibus
GFP: Green fluorescent protein
GSEA: Gene Set Enrichment Analysis
HCC: Hepatocellular carcinoma
HERH: Highly expressed lncRNA in recurrent HCC
lncRNA: Long non-coding RNA
LT: Liver transplantation
miR/miRNA: MicroRNA
MRE: miRNA response element
ncRNA: Non-coding RNA
qRT-PCR: Quantitative reverse transcription - polymerase chain reaction
RB: Retinoblastoma
RIP: RNA immunoprecipitation
siR/siRNA: Small interfering RNA
TF: Transcription factor
