# Supplementary material for: Long non-coding RNAs HERH-1 and HERH-4 facilitate cyclin A2 expression and accelerate cell cycle progression in advanced hepatocellular carcinoma
Source: BMC Cancer. 2021 Aug 26;21:957. doi: 10.1186/s12885-021-08714-7 (PMC8390207; doi:10.1186/s12885-021-08714-7)
Supplement: Supplementary file 2 — Additional file 2. [file 12885_2021_8714_MOESM2_ESM.docx]

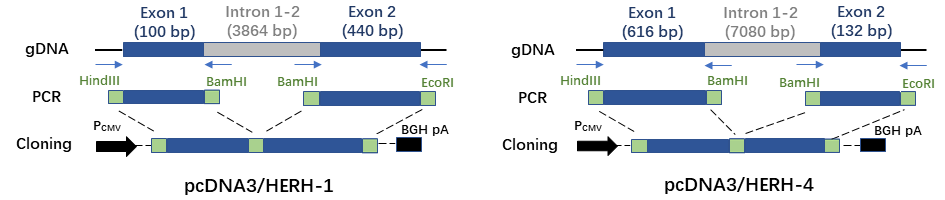


**Figure S1.** Construction of the lncRNA overexpression plasmids.


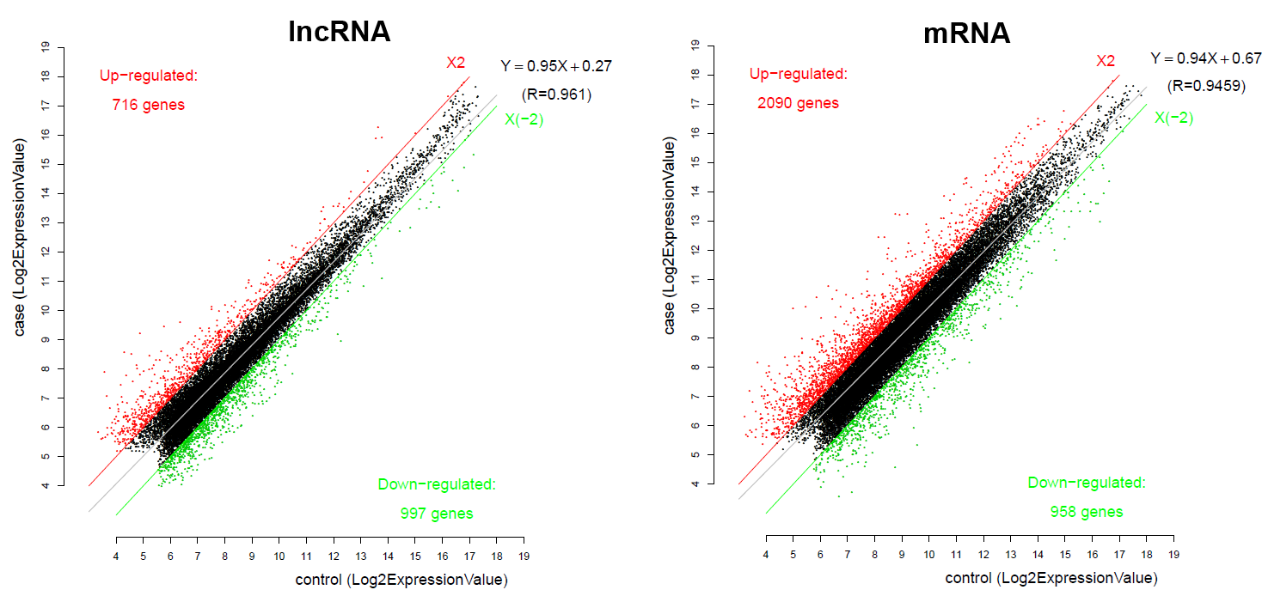


**Figure S2.** Scatter plots of the microarray data (recurrent vs primary).


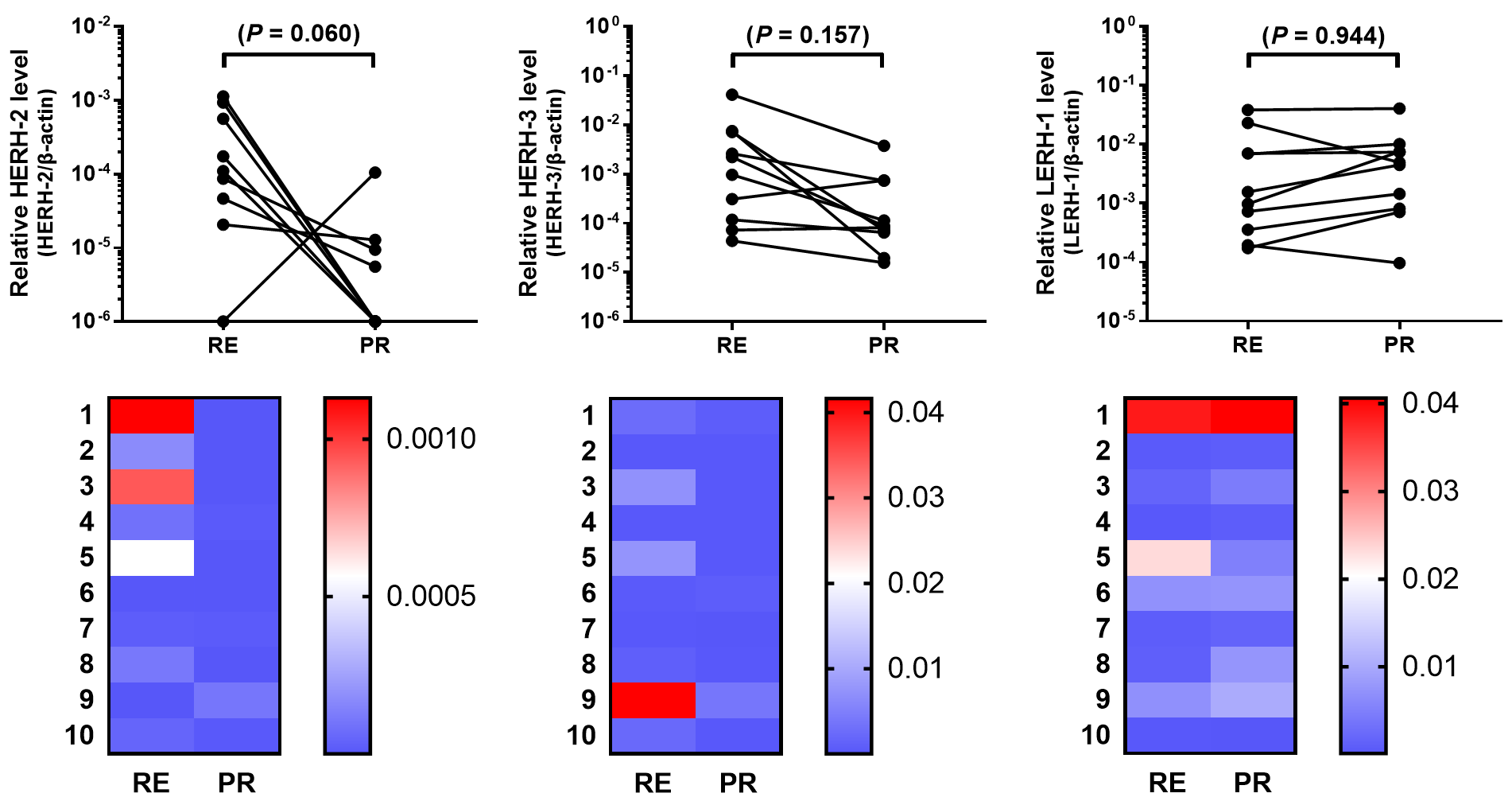


**Figure S3.** The level of the three not-researched lncRNAs (HERH-2, HERH-3 and LERH-1) in HCC recurrent (RE) and primary (PR) tissues were detected by qRT-PCR.


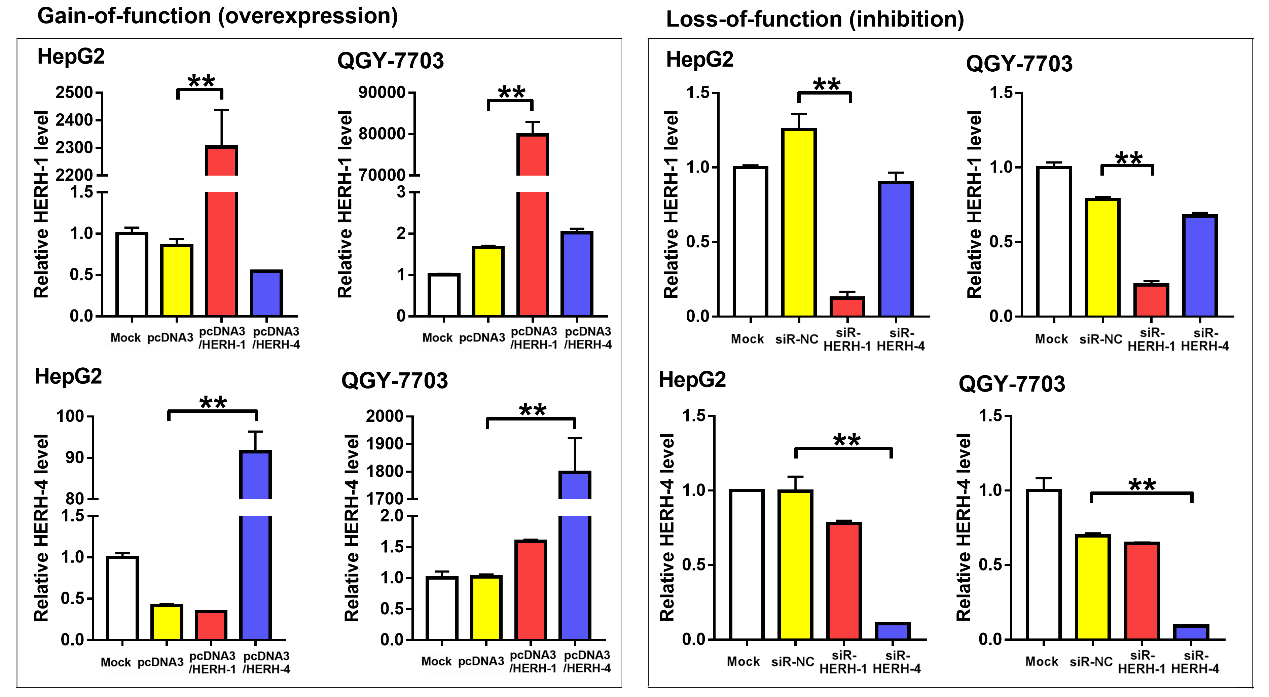


**Figure S4.** Artificial intervention of HERH-1 and HERH-4 levels in HCC cell lines. The HERH-1/4 levels were detected by qRT-PCR. ***P* < 0.01; NC, negative control.


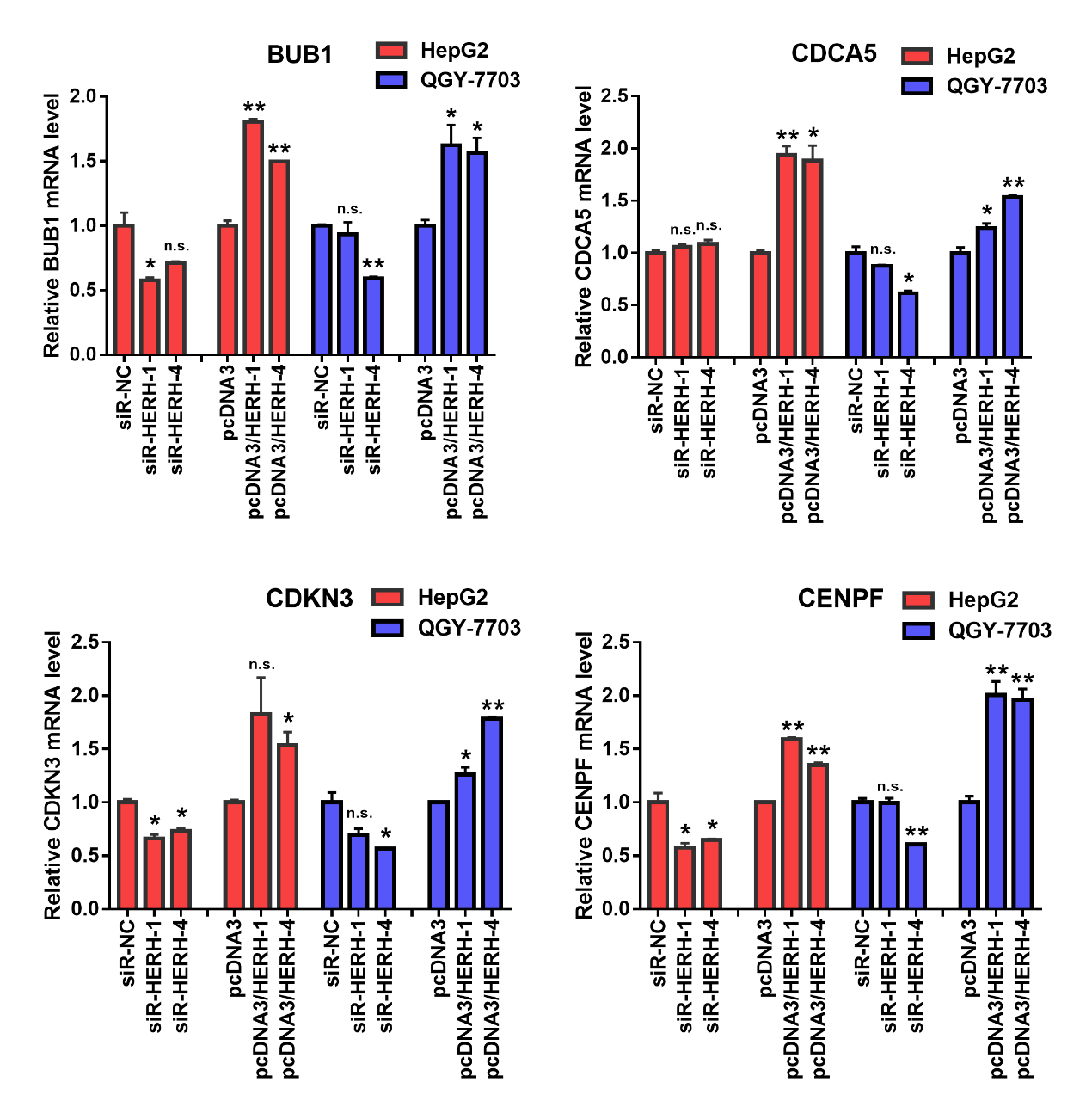


**Figure S5.** Regulation of the selected cell cycle associated genes by siRNA (siR) mediated knockdown or eukaryotic expression plasmid (pcDNA3) induced expression of HERH-1/4 in HCC cell lines. The mRNA levels of BUB1, CDCA5, CDKN3 and CENPF were detected by qRT-PCR. **P* < 0.05; ***P* < 0.01; NC, negative control; n.s., not significant.


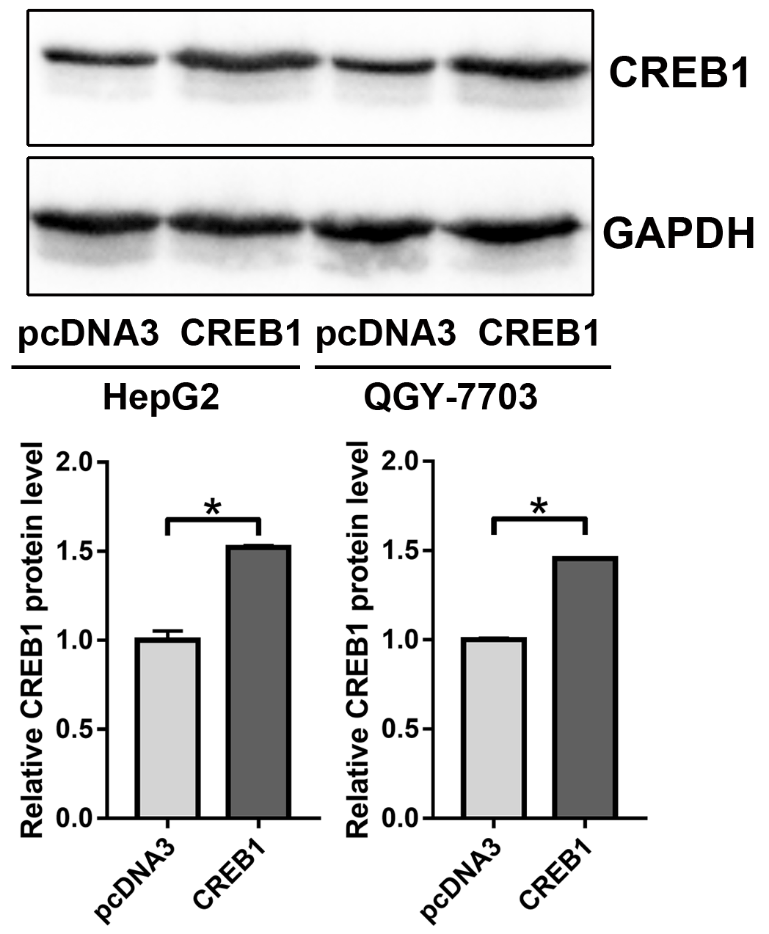


**Figure S6**. Plasmid based CREB1 protein overexpression in HCC cell lines. The CREB1 protein level was detected by Western blot assay. Full-length blots are presented in Fig. S8h. **P* < 0.05


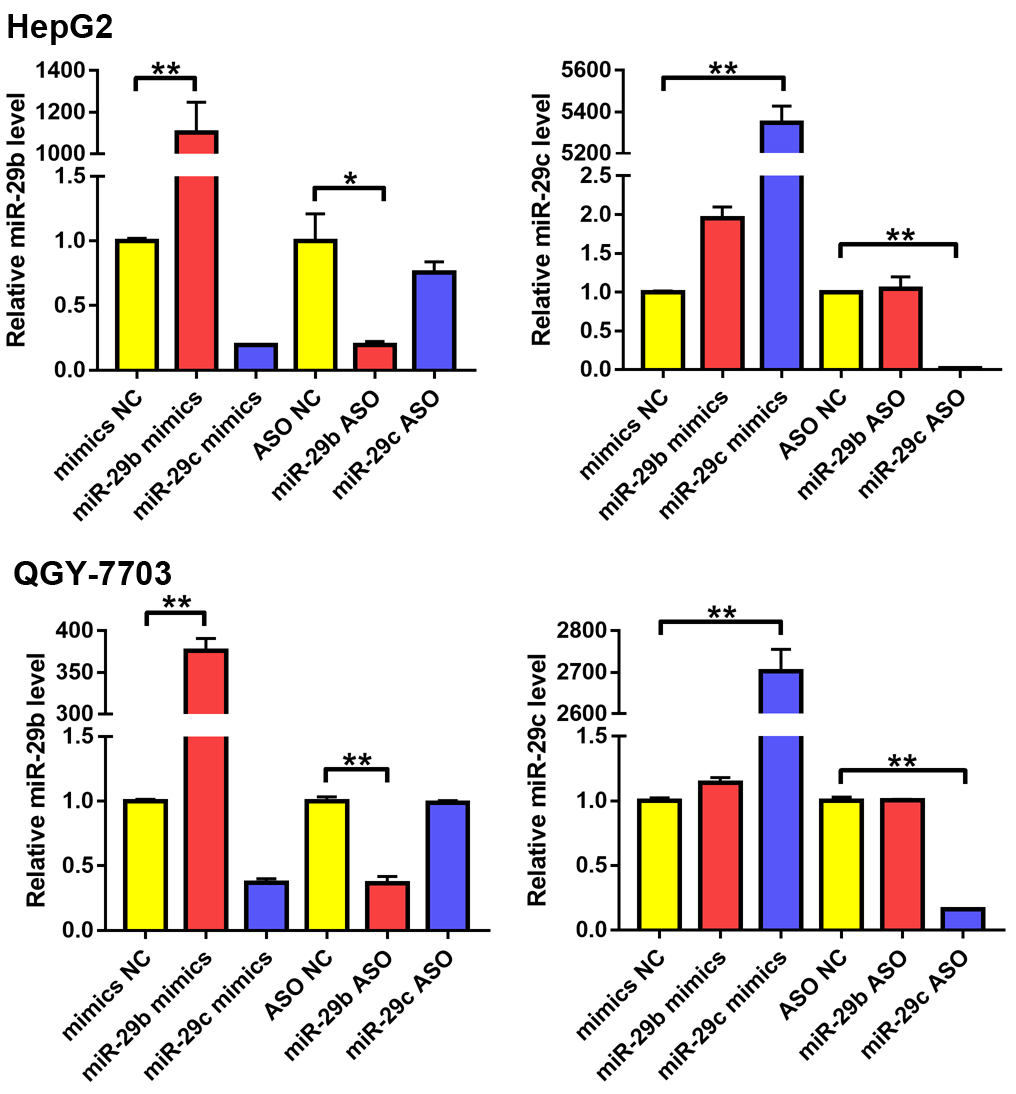


**Figure S7.** Intervention of miR-29b/c in HCC cell lines. The miR-29b/c levels were detected by qRT-PCR. ASO, antisense oligonucleotide; NC, negative control; **P* < 0.05; ***P* < 0.01


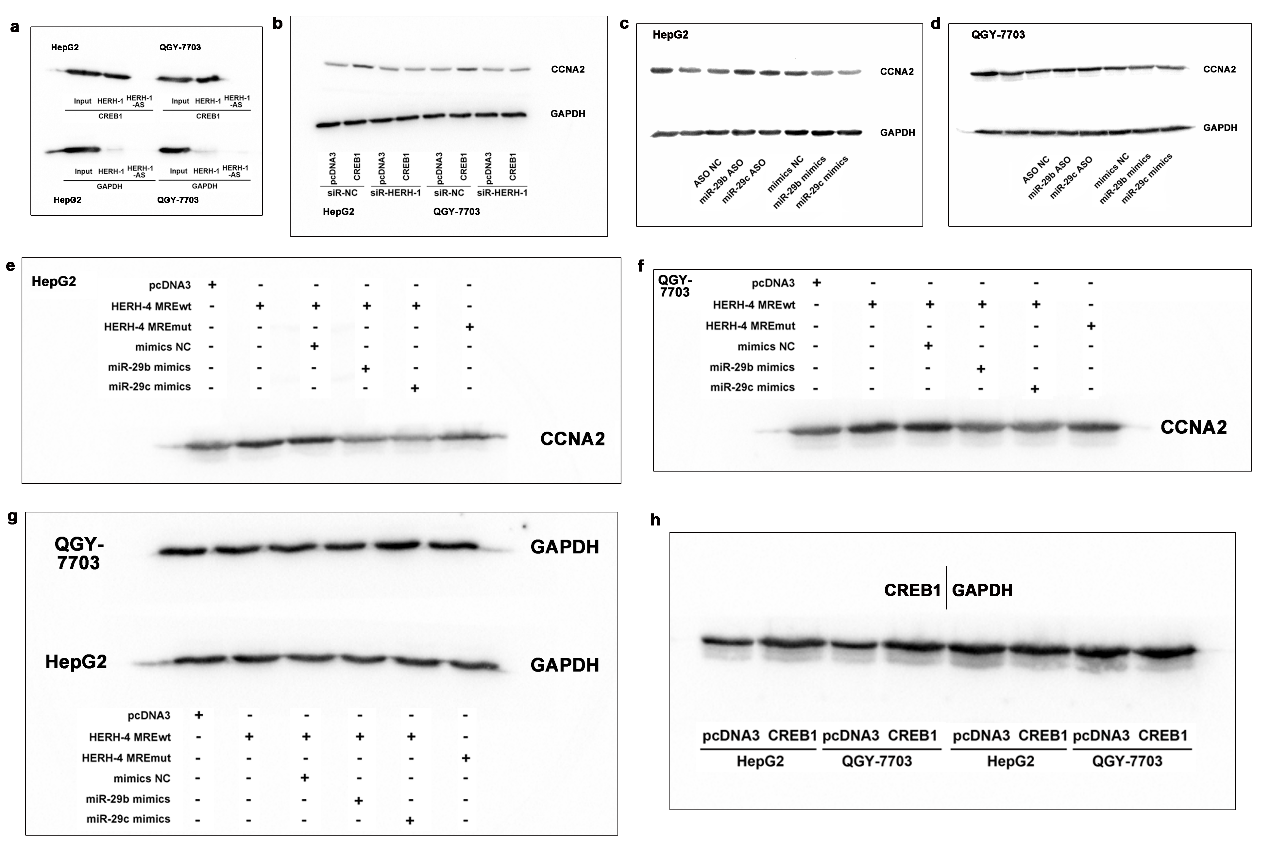


**Figure S8.** The uncropped full-length Western blots for Fig. 3h (a), Fig. 4b (b), Fig. 5e (c, d), Fig. 5g (e–g), and Fig. S6 (h) are shown.


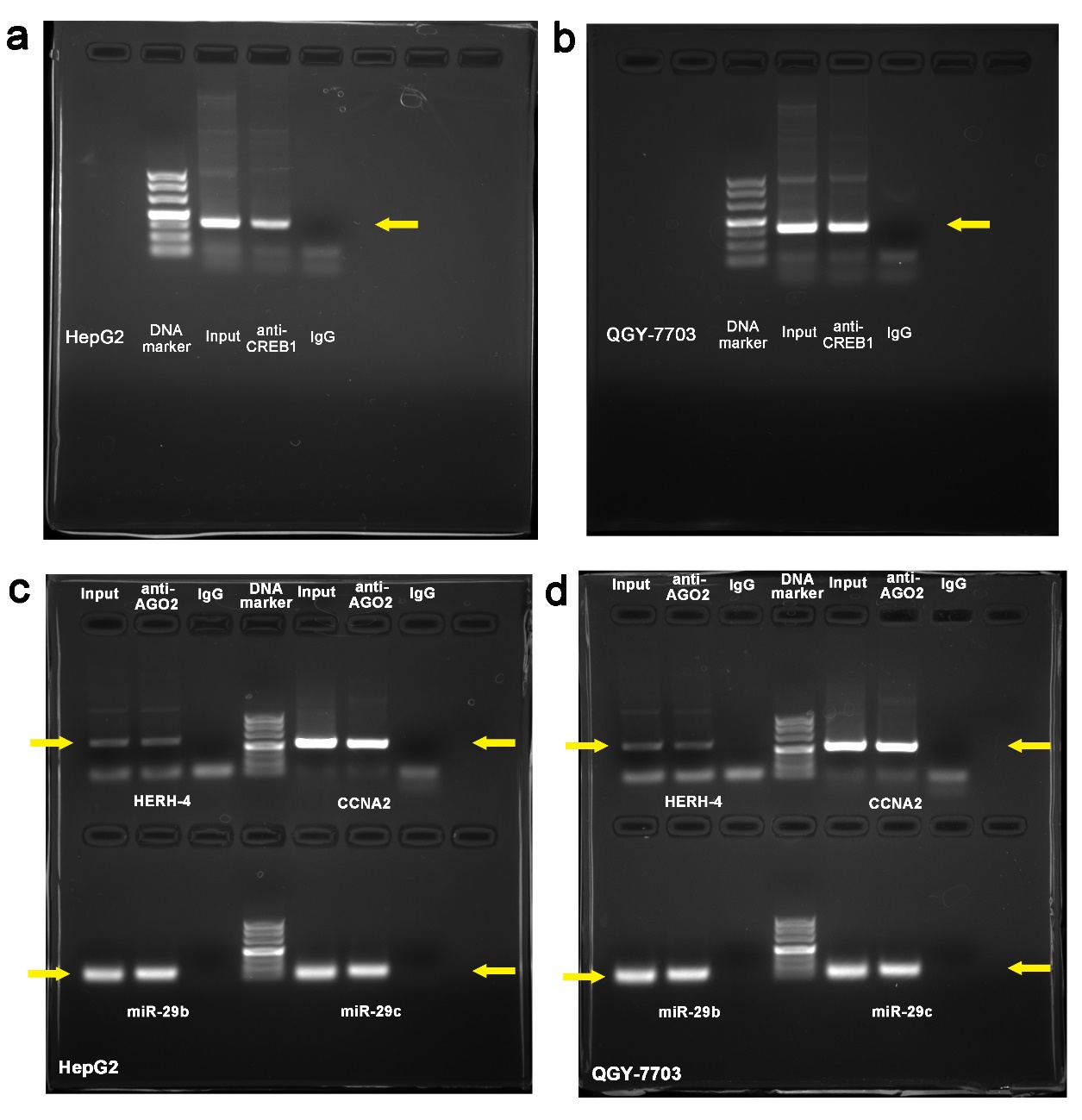


**Figure S9.** The uncropped full-length DNA electrophoresis gels for Fig. 3i (a, b) and Fig. 5i (c, d) are shown. The arrows indicate the target bands.
